# Towards precision oncology discovery: four less known genes and their unknown interactions as highest-performed biomarkers for colorectal cancer

**DOI:** 10.1038/s41698-024-00512-1

**Published:** 2024-01-20

**Authors:** Yongjun Liu, Yuqing Xu, Xiaoxing Li, Mengke Chen, Xueqin Wang, Ning Zhang, Heping Zhang, Zhengjun Zhang

**Affiliations:** 1 Department of Laboratory Medicine and Pathology, University of Washington Medical Center, Seattle, WA USA; 2 Department of Statistics, University of Wisconsin-Madison, Madison, WI USA; 3 State Key Laboratory of Oncology in South China, Sun Yat-sen University Cancer Center, Guangzhou, Guangdong China; 4 Department of Statistics and Finance, University of Science and Technology of China, Hefei, China; 5 Department of Gastroenterology, First Affiliated Hospital of Sun Yat-sen University, Guangzhou, China; 6 Yale School of Public Health, Yale University, New Haven, CT USA; 7 Department of Biostatistics and Medical Informatics, School of Medicine and Public Health, University of Wisconsin-Madison, Madison, WI USA; 8 School of Economics and Management, and MOE Social Science Laboratory of Digital Economic Forecasts and Policy Simulation, University of Chinese Academy of Sciences, Center for Forecasting Sciences, Chinese Academy of Sciences, Beijing, China

**Keywords:** Cancer genomics, Cancer genomics

## Abstract

The goal of this study was to use a new interpretable machine-learning framework based on max-logistic competing risk factor models to identify a parsimonious set of differentially expressed genes (DEGs) that play a pivotal role in the development of colorectal cancer (CRC). Transcriptome data from nine public datasets were analyzed, and a new Chinese cohort was collected to validate the findings. The study discovered a set of four critical DEGs - CXCL8, PSMC2, APP, and SLC20A1 - that exhibit the highest accuracy in detecting CRC in diverse populations and ethnicities. Notably, PSMC2 and CXCL8 appear to play a central role in CRC, and CXCL8 alone could potentially serve as an early-stage marker for CRC. This work represents a pioneering effort in applying the max-logistic competing risk factor model to identify critical genes for human malignancies, and the interpretability and reproducibility of the results across diverse populations suggests that the four DEGs identified can provide a comprehensive description of the transcriptomic features of CRC. The practical implications of this research include the potential for personalized risk assessment and precision diagnosis and tailored treatment plans for patients.

## Introduction

Colorectal cancer (CRC) is a significant public health issue, being one of the most prevalent human malignancies worldwide and the second leading cause of cancer-related deaths^1–3^. While surgical resection, chemoradiation, and immunotherapy have advanced, they remain inadequate in many cases. Moreover, the incidence of CRC is increasing in younger individuals, particularly in the United States and other countries^4–6^. Genetic predisposition plays a crucial role in the development of CRC, with hereditary and sporadic causes accounting for a significant proportion of cases^2,6,7^. The etiology of CRC can be broadly classified into two categories: hereditary or sporadic. Hereditary CRC accounts for 10−15% of the overall incidence and is attributable to mutations in APC or DNA mismatch repair genes. Sporadic CRC is more frequent, representing >80% of CRCs, and is characterized by chromosomal instability, microsatellite instability (MSI), or CpG island methylation^6,7^.

Over the past decades, many transcriptomic studies have been performed which have shed light on the molecular mechanisms underlying CRC development, with a large number of genes being identified as differentially expressed between tumor and nontumor tissues^8–15^. At the molecular level, CRC are classified into four consensus molecular subtypes (CMS), each of which is characterized by distinct expression profiles of oncogenic/tumor suppressive genes and pathways, mutation states of particular genes, MSI, and clinical outcomes^16,17^, however their clinical utility remains to be validated.

So far, most transcriptomic studies have used traditional analytical approaches which rely on fold changes of individual genes between tumor and control tissues or pathway enrichment analysis based on current knowledge of genes and biological processes^11,18–20^. As a result, the number of genes/transcripts reported is large and it is uncertain which of them plays a critical role in cancer identification and classification. Furthermore, gene-gene interactions were not well addressed in traditional analytical models. Thus, there is a need to develop novel analytical methods to identify critical DEGs with high sensitivity and specificity. Recent advances in the machine learning community have shown great promise for applying new methods to improve cancer identification/classification and have demonstrated superior performance over traditional methods^21–23^.

In this study, we applied a newly proven and powerful machine-learning method to identify a parsimonious subset of critical differentially expressed genes (DEGs) for CRC. Our method is based on the max-logistic competing structure, which takes into account the competing relationships among genes in predicting the outcome variable, including gene-gene interactions, a feature not captured by traditional analytical models^21–23^. We analyzed ten transcriptome profiling datasets, including nine public datasets and one separate transcriptome dataset collected from a Chinese population. Using the max-logistic competing risk factor models, we identified four critical DEGs, namely, CXCL8 (C-X-C Motif Chemokine Ligand 8), PSMC2 (Proteasome 26S Subunit, ATPase 2), APP (Amyloid Beta Precursor Protein), and SLC20A1 (Solute Carrier Family 20 Member 1), that demonstrated the highest sensitivity, specificity, and robustness for identifying CRC, compared to the existing literature. Furthermore, the results are interpretable and reproducible across different studies of diverse human populations. Our study represents a significant advancement in the identification of critical genes for CRC and demonstrates the potential of interpretable machine learning in cancer identification and classification. We note that many existing machine learning approaches lack interpretability and often exhibit limited robustness across diverse cohorts. Thus, our findings can be considered as valuable contributions to precision oncology, providing insights that may have practical applications in the field of CRC precision medicine.

## Results

### Identification of critical DEGs

Using the data described in Table 1 and Section Data Availability, we identified four critical DEGs, namely, CXCL8, PSMC2, APP, and SLC20A1. We note that PSMC2 has been linked to CRC cancers, but its interactions with other genes haven’t been reported. The other three genes are less known in the CRC literature though they have been listed in the literature. We will discuss further of these four genes.

**Table 1:** Basic information of the nine public datasets and one vadilating dataset.

Dataset [Reference]	Source	Gene expression platform	Sample size	Population	Tumor stage
1^46^	TCGA	RNA-seq	288 COAD samples and 41 normal controls	North American cohort	Stages 1−4
2^46^	TCGA	RNA-seq	471 COAD samples and 41 normal controls	North American cohort	Stages 1−4
3^11^	NCBI GEO GSE39582	RNA-seq	566 CRC samples and 19 normal controls	European cohort	Stages 1−4
4^47^	NCBI GEO GSE9348	Microarray	70 CRC samples with 12 healthy controls	Han Chinese cohort from Singapore	Stage 1
5^48^	NCBI GEO GSE18105	Microarray	94 CRC samples, and 17 paired sample from tumor and adjacent tissue	Japanese cohort	Stages 2 and 3
6^49^	NCBI GEO GSE41258	Microarray	186 primary CRC and 54 normal colon	Israel cohort	Stages 1−4
7 ^[NA]^	Validating dataset	Real-time quantitative RT-PCR	45 CRC samples and 47 normal controls	Han Chinese cohort from China	Stages 1−4
8^46^	TCGA	RNA-seq	167 READ samples and 10 normal controls	North American cohort	Stages 1−4
9^50^	NCBI GEO GSE103512	RNA-seq	57 primary CRC and 12 normal controls	North American cohort	Stages 1−4
10^17^	NCBI GEO GSE156451	RNA-seq	72 primary CRC and 72 normal controls	Chinese cohort	Stages 1−4

### Identification of classifiers based on the four critical DEGs

Each of the CFs (competing factors) that are in competition with each other (${{CF}}_{{\boldsymbol{i}}}{\boldsymbol{,}}\,{\boldsymbol{i}}{\boldsymbol{=}}{\boldsymbol{1}}{\boldsymbol{,}}\,{\boldsymbol{2}}{\boldsymbol{,}}\,{\boldsymbol{3}}{\boldsymbol{)}}$ can be expressed as a linear combination of gene expressions from critical DEGs. The final classifiers used were the combination of these three competing factors, as presented in Table 2. The risk probability was calculated by applying the logistic function of ***exp***(*Data_****i_****CF*_*max*_)**/(1** **+** ***exp*(***Data_****i_****CF*_*max*_)) for the combined classifiers in each dataset, and of ***exp***(*Data****_i_****CF*_***j***_)**/**(**1** **+** ***exp***(*Data_****i_****CF*_***j***_)) for each individual classifier ***i*** **= 1, 2, 3,** ***j =*** **1, 2, 3**. Data_*i_*CF*j* represents one of the gene-CRC relationships and reflects how genes interact with each other. There may be multiple gene expression combinations (e.g., ***j*** **= 1, 2, 3**) for a particular patient, representing the competing risk factors for that patient. ${Data\_}{\boldsymbol{i\_}}{{CF}}_{\max }$, ***i*** **= 1, 2, 3**, represents the combined maximum of linear competing factors of the *i*th dataset.

**Table 2:** The four critical DEGs and the classifiers identified in the 10 datasets.

Dataset	Data source	Tumor	Non-tumor	Classifier	Intercept	APP	CXCL8	PSMC2	SLC20A1	Accuracy	Sensitivity	Specificity
1	TCGA329	288	41	CF1	−90.3645	2.8598		5.5149	−0.8795	86.02%	85.07%	92.68%
				CF2	−26.5288		0.5692	3.1516	−1.0474	95.74%	96.18%	92.68%
				Max						97.87%	98.61%	92.68%
				CF_Stage 1	−6.3439		0.8358			85.71%	90.70%	80.49%
2	TCGA512	471	41	CF1	9.7471	3.8373	3.4912		−14.1533	96.29%	97.66%	80.49%
				CF2	−8.5448		2.6917	17.7379	−13.4588	97.27%	98.73%	80.49%
				Max						98.24%	99.79%	80.49%
				CF_Stage 1	−11.1078		5.7651			88.50%	95.83%	75.63%
3	GSE39582	566	19	CF1	−44.7356	1.3067		3.133		89.40%	89.22%	94.74%
				CF2	−47.3452	9.0331			−5.8066	26.15%	23.85%	94.74%
				CF3	−28.1527		3.4292	4.936	−4.6261	98.46%	98.76%	89.47%
				Max						99.32%	99.82%	84.21%
				CF_Stage 1	−4.0848		0.5331			90.38%	96.97%	78.95%
4	GSE9348	70	12	CF_Stage 1	−0.2712		0.0011			100%	100%	100%
5	GSE18105	94	17	CF1	−3.1785	−4.3298	0.4519	4.305	−1.9872	99.10%	100%	94.12%
6	GSE41258	186	54	CF1	−1.7889	0.6933	11.5492		−0.1163	94.58%	95.70%	90.74%
				CF_Stage 1	−2.9511		0.0238			91.46%	89.29%	92.59%
7	Self-collected	45	47	CF1	0.4565	−1.6293				53.26%	4.44%	100%
				CF2	−0.4722			1.3083	−1.6565	64.13%	35.56%	91.49%
				CF3	−1.963	−1.677	2.9094			82.61%	77.78%	87.23%
				Max						88.04%	91.11%	85.11%
8	TCGA_READ	167	10	CF1	6.9125		3.3834		−5.007	72.32%	71.26%	90.00%
				CF2	−24.4459	−4.8627		13.8489		90.40%	91.02%	80.00%
				Max						97.18%	98.20%	80%
9	GSE103512	57	12	CF1	−16.337	−4.9005		9.4632	−3.6135	85.51%	84.21%	91.67%
				CF2	−34.282		3.456	2.9659	−2.0026	85.51%	84.21%	91.67%
				Max						98.55%	100%	91.67%
10	GSE156451	72	72	CF1	−11.7722	−0.0677		3.1961	−0.8797	90.97%	90.28%	91.67%
Total	2341	2016	325							**97.74%**	**98.81%**	**91.08%**

The final classifiers are combined classifiers of individual competing factors.

In the first, second, eighth, and ninth datasets, two classifiers, CF_1_ and CF_2_, had sufficiently high powers to identify CRC patients, and additional CFs were not required. In the third and seventh datasets, however, three classifiers (CF_1_, CF_2_ and CF_3_) were needed to accurately predict the presence of CRC tumors, due to the low sensitivity of CF_2_ in those datasets. In the remaining datasets, only one classifier (CF_1_) was needed to achieve the highest sensitivity and specificity in identifying CRC.

**Fig. 1:**
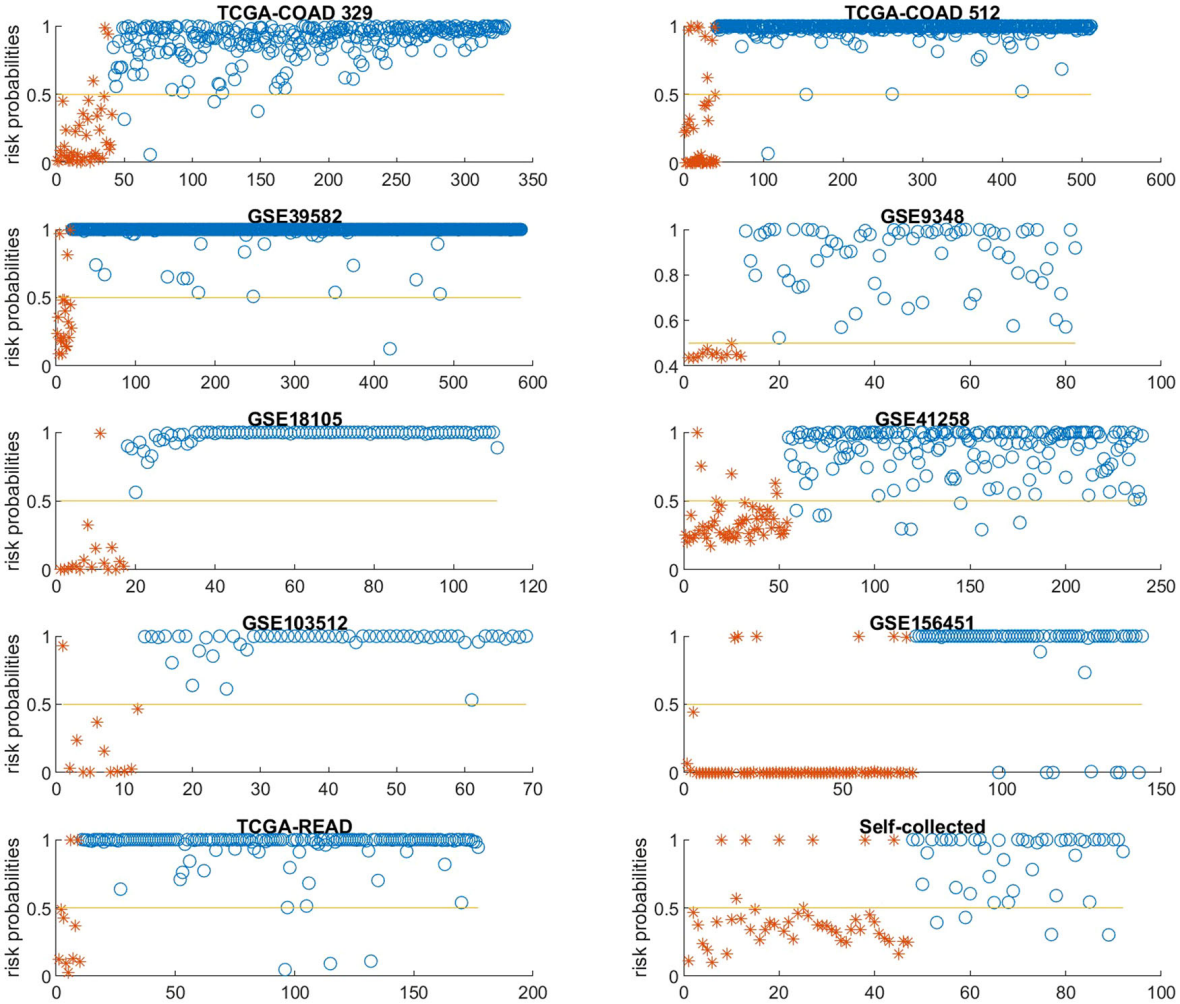
Probabilities of risk estimated by final classifiers for all ten cohorts. CRC samples and normal controls are designated by asters and circles, respectively. A horizontal line at 0.5 (50%) probability threshold is shown in each panel.

**Fig. 2:**
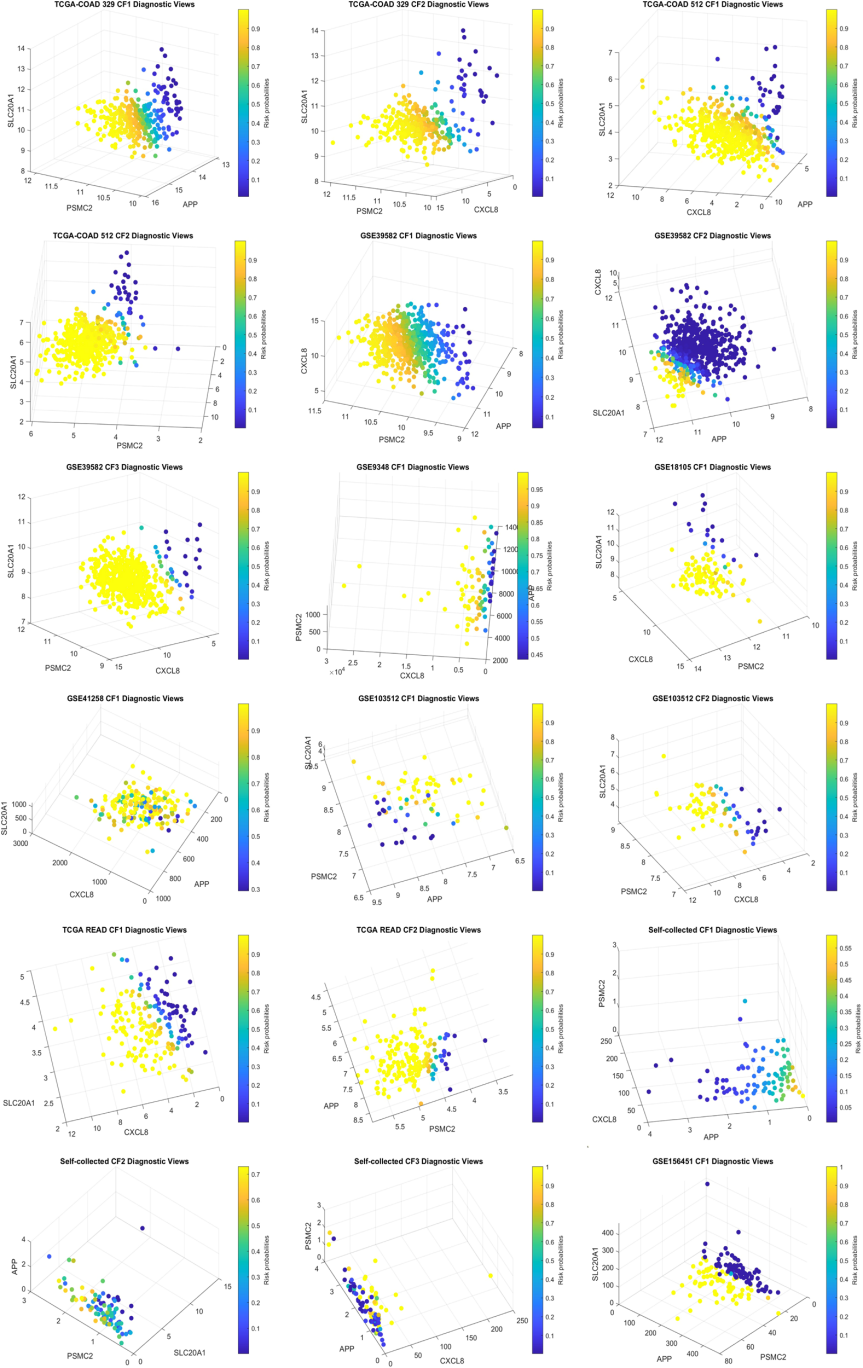
Diagnostic Views of classifiers in each dataset. Gene expression values and their combination effects with different strengths are shown in each plot. The fourth dimension represents the risk probabilities, providing a simple way to identify patients with a high risk of CRC.

For illustration purposes, Fig. 1 displays the risk probabilities estimated by the final classifiers in all datasets. Figure 2 is four-dimensional plots that visualize the signature patterns formed by each classifier in all datasets. Each plot demonstrates how the genes create signature patterns in a geometry space, with the classifiers separating yellow from blue and green colors. Notably, these signature patterns are unique to the four specific genes utilized and cannot be replicated by arbitrarily selecting three different genes.

**Fig. 3:**
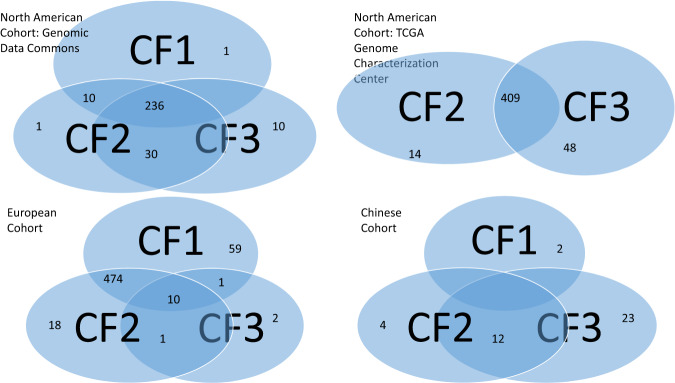
Venn Diagrams for Four Datasets. Venn diagrams displaying the classification of CRC patients into distinct subgroups for selected cohorts (Venn diagrams for Datasets 8 and 9 are similar to Datasets 1 and 2).

Figure 3 depicts a Venn diagram that showcases the patient subgroups, which were classified by the classifiers in the first three cohorts and the validating Chinese cohort. The results demonstrate that in the first and second cohorts, CRC patients were categorized into three distinct subgroups based on the classifiers mentioned above. The first subgroup included patients who were detected only by CF_1_, while the second subgroup included patients who were only detected by CF_2_. The third subgroup comprised patients who were identified by CF_1_ and CF_2_ simultaneously (as seen in Fig. 3). In the third cohort, patients were classified into seven subgroups based on the classifiers CF_1_, CF_2_, and CF_3_. Similarly, the validating Chinese cohort also had seven distinct subgroups. However, in datasets 4, 5, 6 and 10, patients were not further categorized, as one classifier had sufficient power to identify CRC patients. This figure highlights the intricate nature of disease status as patients were categorized into different subgroups based on their gene-gene interactions at the genomic level. Such categorization would be valuable in determining the effectiveness of CRC diagnosis, prognosis, and management. We note applying other machine-learning and AI approaches cannot identify subtypes of CRC.

Table 2 displays the coefficients associated with the classifiers in all ten datasets. It demonstrates the highest performance of the CF_max_ classifier in discriminating between tumor and nontumor samples, with an overall sensitivity of over 98%, specificity of over 80%, and accuracy of over 94%. Furthermore, the combined use of CF_1_, CF_2_, and CF_3_ improved the ability to detect cancer. Notably, in dataset 4, which comprised early-stage CRC cases in a Han Chinese population, the CXCL8 classifier alone achieved 100% sensitivity, specificity, and accuracy in identifying CRC. Therefore, we conducted focused analyses on early-stage CRC (stage 1) in other datasets. The results revealed that CXCL8 alone demonstrated high sensitivity (**>**89%), specificity (**>**75%), and accuracy (**>**85%) in identifying stage 1 CRC in datasets 1, 2, 3, and 6. However, focused analysis was not conducted on the remaining datasets, as there were not enough cases of early-stage CRC.

The classifiers in Table 2 were used to determine the risk probability of CRC based on the direction and absolute value of the gene expression coefficient. A positive coefficient indicated that higher gene expression was associated with a higher risk probability of CRC, while a negative coefficient indicated that lower gene expression was associated with a higher risk probability of CRC. For instance, in datasets 1, 2, 3, and 6, a decrease in APP gene expression was associated with a reduced risk probability of CRC, whereas in datasets 5, 7, 8, 9, and 10, APP showed the opposite direction, indicating a different effect of this gene in White and Asians. SLC20A1 was consistently associated with an increased risk probability of CRC across all datasets, indicating that its expression was suppressed in diverse CRC patient populations. Additionally, CXCL8 and PSMC2 were consistently associated with a reduced risk probability of CRC across all datasets, suggesting that their decreased expression was protective against CRC development. The different signs of APP in different classifiers suggest that the relationship of a single human gene with disease status can be nonlinearly correlated, and its interaction with other genes can be either positively or negatively correlated. We note that the existing CRC literature never reported such interpretations.

Notably, positive coefficients were observed for CXCL8 and PSMC2 across all datasets, indicating their central roles in gene-gene interactions and as competing risk factors for CRC development. The varying coefficients of APP in different classifiers suggest that its relationship with CRC is non-linearly correlated and can be modulated by other genes.

For the purpose of illustration, a subset of gene expression values for the four critical DEGs is shown in Table 3. The complete datasets, including original gene expression values and calculated risk probabilities, are available online.

**Table 3:** Gene expression values, competing factors, and risk probability in a portion of the samples for selected cohorts.

Sample ID	Sample type	APP	CXCL8	PSMC2	SLC20A1	CF_1_	CF_2_	CF_3_	CFmax	Pmax
Dataset 1 (North American cohort)										
TCGA-AA-3522-11	0	14.46	2.57	10.12	12.36	−4.05384	−6.10284		−4.05384	0.01706
TCGA-AA-3518-11	0	14.71	2.84	9.95	12.12	−4.08397	−6.24816		−4.08397	0.016562
TCGA-DM-A1DA-01	1	13.32	5.64	10.92	9.79	−0.68019	0.829748		0.829748	0.696302
TCGA-AD-6965-01	1	14.11	5.66	11.10	10.39	2.072846	0.802252		2.072846	0.888236
Dataset 2 (North American cohort)										
TCGA-AA-3511-11A	0	7.865804	0.840519	4.230288	3.752095	−1.66003	−1.22637		−1.22637	0.226818
TCGA-AA-3517-11A	0	7.346597	0.735178	4.032976	6.230919	−9.56825	−9.26049		−9.26049	9.51E-05
TCGA-AZ-4323-01A	1	6.716108	5.586939	4.427988	3.826772	4.067608	4.417109		4.417109	0.988075
TCGA-AA-3971-01A	1	7.566649	4.029144	5.304325	3.394695	5.079653	8.352782		8.352782	0.999764
Dataset 3 (European cohort)										
1353	0	10.90443	4.798452	9.339269	9.580673	−1.15919	−5.59369	−9.92037	−1.15919	0.238814
1354	0	10.95167	8.181189	9.176233	9.897567	−1.91304	−6.76058	−0.59102	−0.59102	0.356402
1961	1	11.19171	9.436294	10.13665	9.334996	1.29651	−2.77754	11.05611	11.05611	0.999984
1962	1	11.05174	11.19743	10.72173	9.063379	2.788005	−3.34919	21.23988	21.23988	1
Dataset 7 (Independent Chinese cohort)										
1359 T	1	1.81	4.63	0.82	0.35	−2.54	0.03	8.46	8.46	1
1360 T	1	0.95	5.1	0.91	0.44	−1.17	−0.01	11.28	11.28	1
1368 N	0	0.45	0.2	1.4	3.19	−0.51	−3.92	−2.15	−0.51	0.38
1369 N	0	2.68	0.23	0.71	0.98	−3.93	−1.16	−5.79	−1.16	0.24

Columns ${{CF}}_{j},j=\mathrm{1,2,3}$ correspond to ${Data}-i-{{CF}}_{j},i=\mathrm{1,2,3},j=\mathrm{1,2,3}$ with the ith dataset in row blocks and the jth competing risk factor. Column CFmax corresponds to ${Data}-i-{{CF}}_{\max }$, $i=\mathrm{1,2,3}$, i.e., the combined maximum of linear competing factors of the ith dataset. Column Pmax corresponds to Data-*i*, $i=\mathrm{1,2,3}$ and the risk probability (truncated to 2 decimal digits for illustration purpose) of a CRC sample evaluated from the *i*th dataset. In the column “Sample type”, value ‘0’ stands for normal control sample, while value ‘1’ stands for CRC.

The risk probability of a sample with CRC can be calculated using the logistic function in Data_*i_*CF*j*. Table 4 shows the patient subgroups defined by individual classifiers and their combinations in each dataset. The table demonstrates that there were at least seven subgroups of CRC patients, each with different genetic characteristics and determinations. For instance, in Dataset 1, four patients were detected by Data-3-CF_1_, one patient by Data-3-CF_2_, 94 patients by Data-3-CF_3_, and 60 patients were detected simultaneously by all three classifiers. This illustrates the heterogeneity of CRC and the potential utility of genetic subtyping for CRC diagnosis, prognosis, and management.

**Table 4:** Subgroups of patients by individual classifiers and their combinations.

Dataset	CF_1_ only	CF_2_ only	CF_3_ only	CF_1&2_ only	CF_1&3_ only	CF_2&3_ only	All CF_1_,CF_2_,CF_3_
1	7	39	NA	238	NA	NA	NA
2	5	10	NA	455	NA	NA	NA
3	4	1	94	1	399	6	60
7	2	4	23	NA	NA	12	NA
8	12	46	NA	108	NA	NA	NA
9	6	4	NA	51	NA	NA	NA

Since only one classifier was defined for datasets 4, 5, 6, and 10, patients were not further subgrouped.

### Analysis in validating Chinese cohort

We evaluated the performance of the four crucial DEGs (CXCL8, PSMC2, APP, and SLC20A1) identified in the aforementioned public datasets in a validating Chinese cohort from Sun Yat-sen University Cancer Center, China. By setting ${\boldsymbol{K}}{\boldsymbol{=}}{\boldsymbol{1}}$ and solving Eq. (5), we obtained the classifiers (Table 2). The classifier achieved an overall accuracy of 88.04%, sensitivity of 91.11%, and specificity of 85.11%. Notably, the coefficient of APP exhibited a different direction (negative) in this Chinese cohort compared to the North American, European, and Israeli cohorts, suggesting diverse effects of this gene in different populations.

### Analysis in validating the consensus molecular subtypes

Given well-documented heterogeneity of CRC, we performed additional analysis to validate the CMS proposed by other groups (source: https://www.cell.com/trends/cancer/pdf/S2405-8033(16)30098-X.pdf). Theoretically, when we have access to CMS subtype information, it is possible to integrate CMS with the subtypes generated by our competing risk classifiers (CF), creating more nuanced subtypes. This amalgamation can offer a deeper insight into CRC pathology. To illustrate, consider a CMS subtype, let’s say CMS1, and our model-defined subtypes CF_i_CF_j_. The combination of (CMS1, CF_i_CF_j_) gives rise to a novel subtype, as it possesses distinct characteristics not found in other subtypes. The dataset GSE156451^17^ included four distinct CMS with divergent biology and clinical behavior. Therefore, we directly fitted our model using the identified four genes and we found the four genes led to high accuracy (accuracy 90.9722%, sensitivity 90.2778%, specificity 91.6667%). The performance is similar to that in the Chinese cohort that we collected for this study (i.e., the seventh dataset). The following table shows its performance within each CMS subtype. We note that there are 7 patients that were listed as “No group” in the published paper^17^. In addition, there are 8 patients whose information was not disclosed in the published paper, and we denote those as “Others” in Table 5.

**Table 5:** Direct check of model performance in CMS subtypes.

CMS subtype	Sample size	Accuracy	Sensitivity	Specificity
1	10 + 10	95%	100%	90%
2	19 + 19	92.11%	94.74%	89.47%
3	13 + 13	92.31%	84.62%	100%
4	15 + 15	93.33%	93.33%	93.33%
Overall 1-4	57 + 57	93.86%	92.99%	94.74%
No group	6 + 6	100%	100%	100%
Others	9 + 9	72.23%	66.67%	77.78%
Overall	72 + 72	90.97%	90.28%	91.67%

We conducted further analysis within each CMS subtype and obtained Table 6.

**Table 6:** Performance of fitting withing each CMS subtype.

CMS subtype	Sample size	Accuracy	Sensitivity	Specificity
1	10 + 10	100%	100%	100%
2	19 + 19	94.74%	94.74%	94.74%
3	13 + 13	96.15%	92.31%	100%
4	15 + 15	96.67%	100%	93.33%
Overall 1-4	57 + 57	96.49%	96.49%	96.49%

From above tables, it is apparent that the identified four genes show greater universality and specialty. When fitting them directly to each CMS subtype, the results are further refined to achieve higher accuracy, which shows the four critical genes contain not only general genomic-level information for CRC but also CMS subtype information.

Looking at group “Others”, the accuracy is much lower than the other four types and “No group”, which suggests these patients’ CRC types are not typical. Once again, this finding reinforces the connection between the four genes and CRC.

It is evident that the classifiers established by the four critical genes have distinct forms, implying the heterogeneity of CRC, which aligns with the CMS theory^24^.

### Results interpretation through heatmaps

**Fig. 4:**
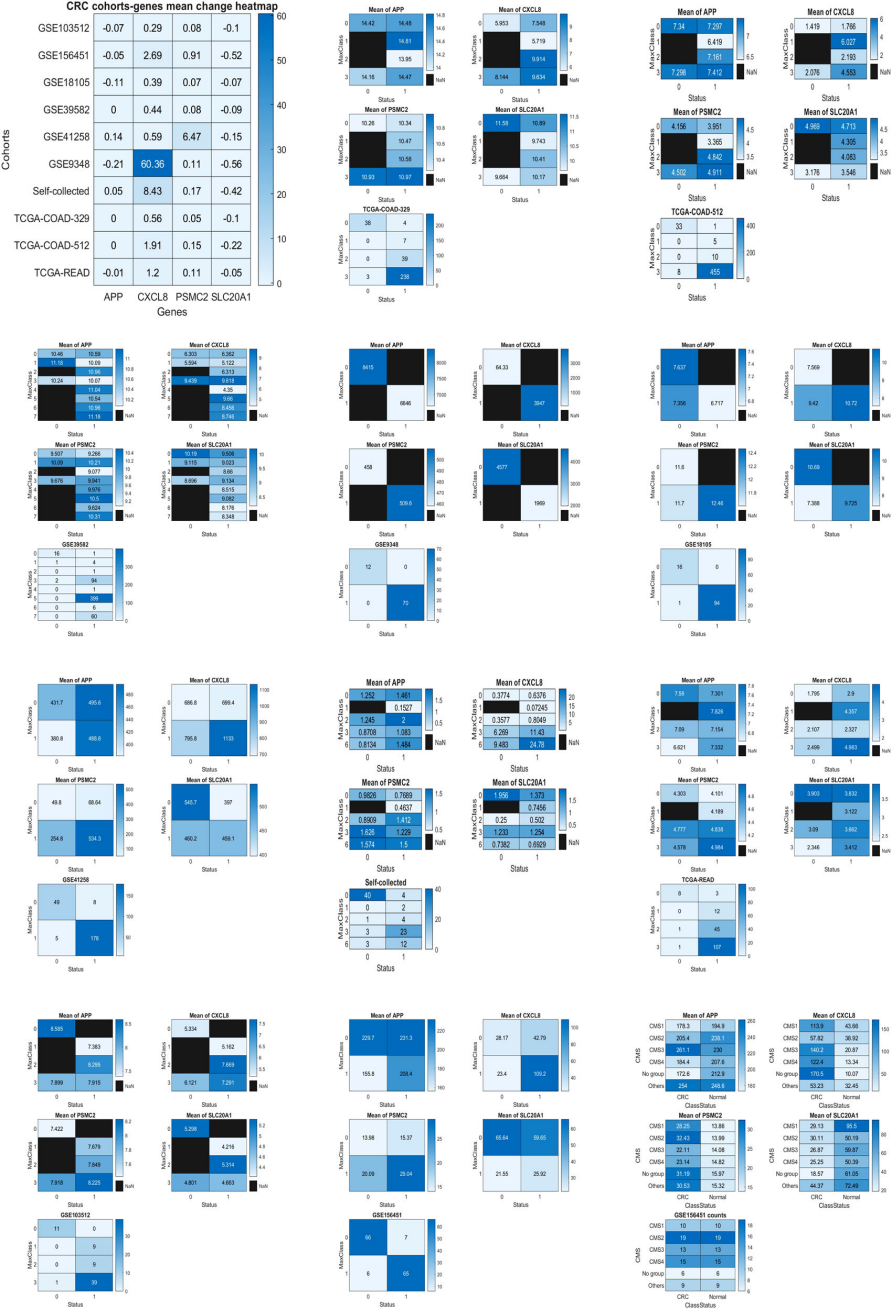
Heatmaps of heteogeneous populations and classification patterns. First row Left: Heatmaps of heteogeneous populations and classification patterns: A heatmap of the selected genes for all cohorts and samples. Middle: For the first dataset TCGA-COAD-329, the mean value of CXCL8 in the normal cell (0,0) is significantly smaller than the mean values of CXCL8 in CRC cells (1,2), (1,3); the mean value of PSMC2 in (0,0) is smaller than all mean values in all other cells; the mean value of SLC20A1 in (0,0) is larger than the mean values in all other cells. This phenomenon confirms CXCL8, PSMC2, and SLC20A1 have essentially important CRC information. Right: For the second dataset TCGA-COAD-512, the mean value of CXCL8 in the normal cell (0,0) is significantly smaller than the mean values of CXCL8 in CRC cells (1,1), (1,2), (1,3); the mean value of PSMC2 in (0,0) is smaller than the mean values of PSMC2 in cells (1,2) and (1,3); the mean value of SLC20A1 in (0,0) is larger than the mean values in all other cells. This phenomenon confirms CXCL8, PSMC2, and SLC20A1 have essentially important CRC information. Second row Left: For the third dataset GSE39582, the mean value of CXCL8 in the normal cell (0,0) is significantly smaller than the mean values of CXCL8 in CRC cells (1,1), (1,3), (1,5), (1,6), (1,7); the mean value of PSMC2 in (0,0) is smaller than the mean values of PSMC2 in cells (1,1) and (1,3-7); the mean value of SLC20A1 in (0,0) is larger than the mean values in all other cells. This phenomenon confirms CXCL8, PSMC2, and SLC20A1 have essentially important CRC information. Middle: For the fourth dataset GSE9348, the mean value of CXCL8 in the normal cell (0,0) is significantly smaller than the mean values of CXCL8 in CRC cells (1,1); the mean value of PSMC2 in (0,0) is smaller than the mean values of PSMC2 in cell (1,1); the mean value of SLC20A1 in (0,0) is twice larger than the mean value in cell (1,1). This phenomenon confirms CXCL8, PSMC2, and SLC20A1 have essentially important CRC information. Right: For the fifth dataset GSE18105, the mean value of CXCL8 in the normal cell (0,0) is smaller than the mean values of CXCL8 in CRC cells (1,1); the mean value of PSMC2 in (0,0) is smaller than the mean values of PSMC2 in cell (1,1); the mean value of SLC20A1 in (0,0) is larger than the mean value in cell (1,1). This phenomenon confirms CXCL8, PSMC2, and SLC20A1 have essentially important CRC information. Third row Left: For the sixth dataset GSE41258, the mean value of CXCL8 in the normal cell (0,0) is significantly smaller than the mean values of CXCL8 in CRC cells (1,1); the mean value of PSMC2 in (0,0) is ten times smaller than the mean values of PSMC2 in cell (1,1); the mean value of SLC20A1 in (0,0) is larger than the mean value in cell (1,1). This phenomenon confirms CXCL8, PSMC2, and SLC20A1 have essentially important CRC information. Middle: For the self-collected dataset, the mean value of CXCL8 in the normal cell (0,0) is significantly smaller than the mean values of CXCL8 in CRC cells (1,2), but 30-80 times smaller than those in (1,3) and (1,6); the mean value of PSMC2 in (0,0) is smaller than the mean values of PSMC2 in cell (1,2), (1,3), (1,6); the mean value of SLC20A1 in (0,0) is larger than the mean value in all other cells. This phenomenon confirms CXCL8, PSMC2, and SLC20A1 have essentially important CRC information. Right: For the eighth dataset TCGA-READ, the mean value of CXCL8 in the normal cell (0,0) is significantly smaller than the mean values of CXCL8 in all CRC cells; the mean value of PSMC2 in (0,0) is smaller than the mean values of PSMC2 in all CRC cells; the mean value of SLC20A1 in (0,0) is larger than the mean value in all CRC cells. This phenomenon confirms CXCL8, PSMC2, and SLC20A1 have essentially important CRC information. Fourth row Left: For the nineth dataset GSE103512, the mean value of CXCL8 in the normal cell (0,0) is significantly smaller than the mean values of CXCL8 in CRC cells (1,2) and (1,3); the mean value of PSMC2 in (0,0) is smaller than the mean values of PSMC2 in all CRC cells; the mean value of SLC20A1 in (0,0) is larger than the mean value in cells (1,1) and (1,3). This phenomenon confirms CXCL8, PSMC2, and SLC20A1 have essentially important CRC information. Middle: For the tenth dataset GSE156451, the mean value of CXCL8 in the normal cell (0,0) is three times smaller than the mean values of CXCL8 in CRC cells (1,1); the mean value of PSMC2 in (0,0) is twice smaller than the mean values of PSMC2 in cell (1,1); the mean value of SLC20A1 in (0,0) is twice larger than the mean value in cell (1,1). This phenomenon confirms CXCL8, PSMC2, and SLC20A1 have essentially important CRC information. Right: For the tenth dataset GSE156451and CMS subtypes, the mean values of CXCL8 in the normal column are significantly twice or much smaller than the mean values of CXCL8 in CRC column with respect to each CMS subtype; the mean values of PSMC2 in normal column are twice of nearly twice smaller than the mean values of PSMC2 in CRC column with respect to each CMS subtype; the mean values of SLC20A1 in normal column are twice larger than the mean values in CRC column with respect to each CMS subtype. This phenomenon confirms CXCL8, PSMC2, and SLC20A1 have essentially important CRC information and CMS subtype information.

We present heatmaps using classes of normal vs. CRC, the subtypes classified by our max-logistic competing classifiers, and CMS subtypes. All plots are displayed in Fig. 4. Here, we briefly summarize what the heatmaps tell about the four critical genes.

The first heatmap is a heatmap of the selected genes for all cohorts and samples. It can be clearly seen that the relative mean changes of CXCL8 and PSMC2 are uniformly positive, while the signs of SLC20A1 are all negative. This observation shows that these three genes contain pathological information of CRC. Also, we can notice that the strengths of changes of CXCL8 are much larger/stronger than the strengths from other genes. This phenomenon suggests CXCL8 responded to CRC symptoms earlier and stronger, which is consistent with our analysis with those datasets of Stage I CRC.

For the first dataset TCGA-COAD-329, the mean value (5.953) of CXCL8 in the normal cell (0,0) is significantly smaller than the mean values (9.914, 9.634) of CXCL8 in CRC cells (1,2), (1,3); the mean value (10.26) of PSMC2 in (0,0) is smaller than all mean values in all other cells; the mean value (11.58) of SLC20A1 in (0,0) is larger than the mean values in all other cells. This phenomenon confirms CXCL8, PSMC2, and SLC20A1 have essentially important CRC information.

For the second dataset TCGA-COAD-512, the mean value (1.419) of CXCL8 in the normal cell (0,0) is significantly smaller than the mean values (6.027, 2.193, 4.553) of CXCL8 in CRC cells (1,1), (1,2), (1,3); the mean value (4.156) of PSMC2 in (0,0) is smaller than the mean values (4.842, 4.911) of PSMC2 in cells (1,2) and (1,3); the mean value (4.969) of SLC20A1 in (0,0) is larger than the mean values in all other cells. This phenomenon confirms CXCL8, PSMC2, and SLC20A1 have essentially important CRC information.

For the third dataset GSE39582, the mean value (6.303) of CXCL8 in the normal cell (0,0) is significantly smaller than the mean values of CXCL8 in CRC cells (1,1), (1,3), (1,5), (1,6), (1,7); the mean value (9.507) of PSMC2 in (0,0) is smaller than the mean values of PSMC2 in cells (1,1) and (1,3-7); the mean value (10.19) of SLC20A1 in (0,0) is larger than the mean values in all other cells. This phenomenon confirms CXCL8, PSMC2, and SLC20A1 have essentially important CRC information.

For the fourth dataset GSE9348, the mean value (64.33) of CXCL8 in the normal cell (0,0) is significantly smaller than the mean value (39.47) of CXCL8 in CRC cells (1,1); the mean value (458) of PSMC2 in (0,0) is smaller than the mean value (509.6) of PSMC2 in cell (1,1); the mean value (4577) of SLC20A1 in (0,0) is twice larger than the mean value (1969) in cell (1,1). This phenomenon confirms CXCL8, PSMC2, and SLC20A1 have essentially important CRC information.

For the fifth dataset GSE18105, the mean value (7.569) of CXCL8 in the normal cell (0,0) is smaller than the mean value (10.72) of CXCL8 in CRC cells (1,1); the mean value (11.6) of PSMC2 in (0,0) is smaller than the mean value (12.46) of PSMC2 in cell (1,1); the mean value (10.69) of SLC20A1 in (0,0) is larger than the mean value (9.725) in cell (1,1). This phenomenon confirms CXCL8, PSMC2, and SLC20A1 have essentially important CRC information.

For the sixth dataset GSE41258, the mean value (686.8) of CXCL8 in the normal cell (0,0) is significantly smaller than the mean value (1133) of CXCL8 in CRC cells (1,1); the mean value (49.8) of PSMC2 in (0,0) is ten times smaller than the mean value (534.3) of PSMC2 in cell (1,1); the mean value (545.7) of SLC20A1 in (0,0) is larger than the mean value (459.1) in cell (1,1). This phenomenon confirms CXCL8, PSMC2, and SLC20A1 have essentially important CRC information.

For the self-collected dataset, the mean value (0.3774) of CXCL8 in the normal cell (0,0) is significantly smaller than the mean value (0.8049) of CXCL8 in CRC cells (1,2), but 30-80 times smaller than those (11.43, 24.78) in (1,3) and (1,6); the mean value (0.9826) of PSMC2 in (0,0) is smaller than the mean values of PSMC2 in cell (1,2), (1,3), (1,6); the mean value (1.956) of SLC20A1 in (0,0) is larger than the mean value in all other cells. This phenomenon confirms CXCL8, PSMC2, and SLC20A1 have essentially important CRC information.

For the eighth dataset TCGA-READ, the mean value (1.795) of CXCL8 in the normal cell (0,0) is significantly smaller than the mean values of CXCL8 in all CRC cells; the mean value (4.303) of PSMC2 in (0,0) is smaller than the mean values of PSMC2 in all CRC cells; the mean value (3.903) of SLC20A1 in (0,0) is larger than the mean value in all CRC cells. This phenomenon confirms CXCL8, PSMC2, and SLC20A1 have essentially important CRC information.

For the ninth dataset GSE103512, the mean value (5.334) of CXCL8 in the normal cell (0,0) is significantly smaller than the mean values (7.669, 7.291) of CXCL8 in CRC cells (1,2) and (1,3); the mean value (7.422) of PSMC2 in (0,0) is smaller than the mean values of PSMC2 in all CRC cells; the mean value (5.298) of SLC20A1 in (0,0) is larger than the mean value in cells (1,1) and (1,3). This phenomenon confirms CXCL8, PSMC2, and SLC20A1 have essentially important CRC information.

For the tenth dataset GSE156451, the mean value (28.17) of CXCL8 in the normal cell (0,0) is three times smaller than the mean value (109.2) of CXCL8 in CRC cells (1,1); the mean value (13.98) of PSMC2 in (0,0) is twice smaller than the mean value (29.04) of PSMC2 in cell (1,1); the mean value (65.64) of SLC20A1 in (0,0) is twice larger than the mean value (25.92) in cell (1,1). This phenomenon confirms CXCL8, PSMC2, and SLC20A1 have essentially important CRC information.

For the tenth dataset GSE156451and CMS subtypes, the mean values of CXCL8 in the normal column are significantly twice or much smaller than the mean values of CXCL8 in CRC column with respect to each CMS subtype; the mean values of PSMC2 in normal column are twice or nearly twice smaller than the mean values of PSMC2 in CRC column with respect to each CMS subtype; the mean values of SLC20A1 in normal column are twice larger than the mean values in CRC column with respect to each CMS subtype. This phenomenon confirms CXCL8, PSMC2, and SLC20A1 have essentially important CRC information and CMS subtype information.

In summary, all ten cohorts demonstrate that CXCL8, PSMC2, and SLC20A1 contain essentially important CRC information.

### Characterization of clinical and pathological features

In order to compare the characteristics of subgroups defined by the classifiers, we analyzed various attributes such as sex, age, histologic grades, and TNM tumor stages (as shown in Table 7). It should be noted that some samples did not have complete clinical or pathological information. Datasets 4, 5, and 6 were excluded from this analysis as the CRC patients in these datasets were identified by only one CF. In the first and second datasets, the majority of CRC patients were identified by CF_(1,2)_, which means either CF_1_ or CF_2_ could determine the cancer status. There was no significant difference in gender distribution among subgroups, and patients were found in almost all age groups. In the second dataset, patients identified by individual CF_1_ or CF_2_ appeared to have a higher frequency of late-stage CRC, but the sample size in these subgroups was small. In the third dataset, CF_3_ was the classifier that identified the majority of patients. There was no significant gender preference between groups, but stages 2 and 3 CRC were more common in subgroups associated with CF_3_.

**Table 7:** Distribution of clinical and pathological characteristics.

Classifiers	Sex		Age at diagnosis (years)					TNM Stage			
	Male	Female	(20,50]	(50,60]	(60,70]	(70,80]	(80,100]	I	II	III	IV
The first dataset (North American cohort)											
CF_1_	4	2	1	0	2	3	0	1	3	1	1
CF_2_	19	19	5	5	14	8	5	4	14	13	5
CF_(1,2)_	131	100	38	49	55	62	26	39	89	62	33
The second dataset (North American cohort)											
CF_1_	1	1	1	0	1	0	0	0	0	2	0
CF_2_	4	4	2	2	3	0	1	0	0	8	0
CF_(1,2)_	229	198	55	73	109	126	62	73	167	115	61
The third dataset (European cohort)											
CF_1_	3	1	1	2	0	1	0	0	1	2	1
CF_2_	0	1	0	1	0	0	0	0	1	0	0
CF_3_	47	47	11	13	27	31	12	9	44	33	8
CF_(1,2)_	1	0	0	0	1	0	0	0	1	0	0
CF_(1,3)_	222	177	55	58	114	122	49	20	184	153	41
CF_(2,3)_	4	2	0	1	3	1	1	0	4	2	0
CF_(1,2,3)_	33	27	9	6	20	15	10	4	28	15	10
The seventh dataset (our Chinese cohort)											
CF_1_	0	2	0	1	0	0	1	0	2	0	0
CF_2_	1	3	1	1	1	0	1	2	0	0	1
CF_3_	13	10	5	3	3	8	4	0	7	11	5
CF_(2,3)_	8	4	0	1	6	3	2	1	7	2	2

## Discussion

Transcriptomic studies are crucial in identifying significant genes and biological pathways associated with CRC. However, current transcriptomic data analyses have limitations. Firstly, the number of genes/transcripts analyzed is significantly larger compared to the number of study samples. Secondly, traditional statistical methods based on gene expression fold change used in most studies lack the power to determine key drivers of cancer development due to a large number of genes analyzed. Lastly, gene-gene interaction effects are not adequately addressed in existing analytical models. As a result, some of the DEGs identified in previous studies may be noise effects or chance findings^25^. In contrast, the newly introduced interpretable machine learning method employed in our study is unique as it considers complex gene-gene interactions and competition, which more closely resemble biological processes underlying cancer development. The proposed method identifies critical DEGs and signatures in heterogeneous cohorts, even on different platforms with varying gene expression values. This method has been successfully applied in our early efforts to identify critical DEGs in lung cancer^26^, breast cancer^27^, and COVID-19^23^. Our current study further supports the power and validity of the method. Importantly, our method stands apart from other artificial intelligence methods that function as a black box since our new method follows a transparent and interpretable formula that can be easily validated.

Our study identified four critical DEGs (CXCL8, PSMC2, APP, and SLC20A1) that demonstrated high performance in identifying CRC in diverse populations and ethnicities, covering a range of tumor stages and histologic grades. These genes may serve as biomarkers that can capture the underlying characteristics of CRC at the transcriptomic level. As such, they may have significant practical implications. Given the high performance of our classifier, we hypothesize that individuals who have higher risk of developing CRC can be identified by the classifiers based on four critical genes. For example, colon biopsy specimens from screening colonoscopy procedures can be sequenced for transcriptomic profiles to identify the individuals with a risk to develop CRC. The colon biopsy specimens may include those random biopsies or colon polyp biopsies without definitive histologic evidence of CRC.

It is important to emphasize that the gene-gene interaction effects in our model are distinct from those commonly used in other experimental designs, such as row-column or chemical-laboratory interactions in industrial and agricultural studies. Our method goes beyond the interaction term in a typical linear regression model, where interactions are often analyzed by multiplying two predictors to construct an additional covariate. In our model, the combination of genes in each CF determines the interaction effects, which can be seen in the coefficient values of each CF. This was observed across all datasets analyzed, indicating the presence of gene-gene and gene-subtype interactions. Furthermore, each classifier identified in our study can serve as a potential marker for CRC, and the effects of these classifiers may be modulated by critical genes such as PSMC2 and CXCL8, which are associated with CRC of different tumor stages. To illustrate this concept, we take two classifiers from Table 2 (TCGA COAD data) as an example:

CF1 − 90.3645 +2.8598*APP +5.5149*PSMC2 −0.8795* SLC20A1

CF2 −26.5288 +0.5692*CXCL8 +3.1516*PSMC2 −1.0474* SLC20A1

Consider these classifiers like assembling a basketball team, where each critical gene corresponds to a player on the team. The various combinations of players determine the team’s scoring ability. In this analogy, the gene combinations in each CF influence our model’s classification performance. A positive coefficient associated with a gene in a CF suggests that the presence of that gene increases the likelihood of classifying a sample as CRC, while a negative coefficient indicates the opposite. For instance, let’s envision a basketball team with five players: PSMC2 as the Point Guard, SLC20A1 as the Shooting Guard, CXCL8 as the Center, APP as the Power Forward, and the fifth player to be determined. The accuracy of these two classifiers is 97.87%, leaving room for one more player. This team has two main scoring combinations: (APP, PSMC2, SLC20A1) and (CXCL8, PSMC2, SLC20A1). The negative sign associated with SLC20A1 suggests that its role is to guard against the opponent’s ball handling time, increasing the team’s scoring probability. For the CXCL8, increasing the ball handling time will increasing team’s scoring probability, so do PSMC2 and APP. For SLC20A1, different coefficients in both combinations mean its interactions with other players in the team are different, which decides the different scoring abilities. Other genes and their associated coefficients can be interpreted similarly. While this analogy simplifies gene interactions, it’s important to note that gene-gene interactions are much more complex. They involve intricate interactions, analogous to player playing time, ball handling, coordinated defense, and the overall dynamics of the entire game, including the spectators in the stadium.

The functional relevance of the four critical DEGs to carcinogenesis has been reported. For example, CXCL8, a member of the CXC cytokine family, is one of the most significantly upregulated chemokines in CRC, contributing to tumor growth, invasion, and metastasis^28–33^. CXCL8 induces cell migration in colon cancer cells, acting as an autocrine growth factor^34–36^. In line with this evidence, our results suggest CXCL8 could be a marker for early-stage CRC. Similarly, PSMC2, a key member of the 19 S RP, has been implicated in the progression of several types of cancer, including ovarian cancer, pancreatic cancer, and osteosarcoma^37–40^. High expression of PSMC2 in CRC is associated with poorer survival, and silencing of PSMC2 is a potential therapeutic strategy for CRC^41^. Interestingly, in our study, decreased APP expression was associated with reduced CRC risk in the US, European, and Israel populations, but a reverse effect was observed in Asian populations (Chinese and Japanese) and rectal cancer. SLC20A1, a sodium-phosphate symporter involved in tumor formation by HeLa cells in xenografted mice^42–45^, has a negative coefficient in all datasets, suggesting its expression is inhibited in CRC, although its significance in CRC is currently unknown. We note that although the literature has sparse reports about these four genes as discussed above, their functions are still less known, and their interactions with each other and other genes are totally unknown due to the limitations of the earlier studying methods. As a result, they cannot be used in precision cancer diagnostics. Our new study is the first to achieve precision cancer diagnostics at the genomic level. We note that (CXCL8, PSMC2, SLC20A1) formed classifiers appeared in all North American CRC Cohorts and a European cohort, but not in Asian cohorts, Israel cohort, and the TCGA READ cohort. Such a phenomenon suggests there is genomic heterogeneity among CRC patients due to their ethnicity and subtypes.

Our method has the advantage of being applicable to gene expression data generated by different platforms, including RNA-seq and microarrays, without requiring batch correction. We note that many existing models cannot handle heterogeneous populations, and data have to be corrected for batch effects, which may make the inference inaccurate. Notably, our classifiers achieved the highest accuracy in cancer detection without adjusting for variables such as tumor stage or ethnicity. However, we acknowledge that some public datasets lack complete clinical and pathological information, which limits our ability to explore the prognostic value of the four DEGs. Nonetheless, our study is unique in its adoption of a novel machine learning approach, which has not been previously used in transcriptomic studies of human malignancies. Additionally, our findings were validated across diverse populations and ethnicities, further highlighting the potential of this approach for cancer research.

This study has a few limitations that should be acknowledged. Firstly, as a retrospective analysis of public datasets, it was not possible to conduct a comprehensive prognosis analysis to determine the prognostic value of the identified critical genes in CRC. However, the four-gene-based classifiers identified in this study exhibit the highest performance, providing a promising starting point for future studies to explore their prognostic potential. Secondly, since clinical diagnosis of CRC currently depends mainly on traditional methods such as colonoscopy and tissue biopsies, it may be challenging to identify clinical significance for the critical DEGs identified in this study. However, examining molecular subtypes based on transcriptomic patterns is crucial to understanding the genetic mechanisms underlying carcinogenesis. Integrating reliable genomic biomarkers like the four-gene-based classifiers into the CRC diagnosis algorithm will enhance the accuracy of disease identification and patient classification, leading to personalized and precision oncology. Finally, our study was based exclusively on gene expression profiles of CRC tissues, and it is unclear whether the four-gene-based classifiers can be used to detect CRC patients in the general population using blood samples. To address this issue, future studies could include cohorts with both CRC tissues and blood samples.

One note pertains to the computational aspect and how to solve Eq. (4) across all cohorts and how to integrate the critical gene criteria. As of the present stage, we have yet to develop a complete, fast, and efficient computer program for this purpose, and it remains an open challenge. In our study, we have adopted a simplified approach discussed in methodology section.

While further experimental studies are needed to validate our findings and confirm the significance of the identified critical DEGs, we employed rigorous criteria to define these genes, and the high accuracy of the classifiers, reaching 100% in some cohorts, suggests that the findings are unlikely to be due to chance (probability less than 10^−11^). Moreover, each of the four genes has been previously reported to be functionally relevant to carcinogenesis, and our study is the first to demonstrate their interaction effects in CRC. These findings provide a solid foundation for future investigations, including gene network analysis, exploring related genes and their functional interactions, and identifying potential causal relationships.

## Methods

### Data acquisition and processing

Table 1 presents an overview of the datasets used in this study, including information on data sources, experimental platforms, sample sizes, populations, and tumor stages. The discovery analyses involved nine publicly available whole-transcriptome profiling datasets, including three from the Cancer Genome Atlas (TCGA) obtained through RNA-seq and six from the Gene Expression Omnibus (GEO) database using the keywords “colon cancer”, “CRC”, and “Homo sapiens,” which were based on mRNA expression (microarray-based). The datasets were collected from a diverse population, including North American and European White, Blacks, Asians, and Jewish. In addition, clinical and pathological information, such as age, sex, TNM tumor stages, and histologic grades, were also obtained when available.

### Analytical methodology

Our approach to building a competing risk factor classifier for CRC involved utilizing the max-logistic regression model. This model offered an advantage over existing models in its ability to provide nonlinear prediction and classification. Our aim was to identify a concise subset of key genes with the highest predictive accuracy. The theoretical basis of the competing risk factor models can be found in previous literature^21,22^. To ensure consistent analysis of DEGs across the nine public datasets and our Chinese validation cohort, we utilized the heterogeneous extension of the max-logistic regression. Starting with each competing risk factor in the max-logistic regression models, we randomly selected three genes from each dataset’s available genes/transcripts. To determine the final model with the highest sensitivity and specificity while using the smallest number of genes, we employed a Monte Carlo method that required extensive computation. Our criteria for defining critical DEGs were stringent.

1. The critical DEG subset should contain no more than 15 genes (such a number has been widely reported in the literature). We note that we only need four in CRC study.
2. The critical DEG subset should exhibit an overall accuracy of at least 95% in at least three distinct study cohorts, with a total of at least 1000 patients/subjects.
3. The critical DEG subset should exhibit an overall accuracy of 100% in at least one study cohort, with a minimum of 10 subjects.
4. At least one gene functions and shows the same sign ( + or -) in each study cohort.
5. The critical DEG subset should demonstrate at least 80% accuracy in any given cohort, with either sensitivity or specificity values exceeding 75%.
6. The competing classifiers should include the smallest number of genes possible.
7. The number of competing classifiers should be minimized to avoid redundancy.

We remark that many published work didn’t report cohort-to-cohort validations, i.e., they cannot satisfy (2), (3) and (5). Many existing machine-learning approaches lack interpretability and didn’t satisfy (4). Most published work couldn’t apply to heterogeneous populations, e.g., the ten cohort studies in this paper.

The basic ideas of competing risk classifiers for heterogeneous populations are briefly described below.

Suppose there are $K$ cohorts with their binary (1 for CRC, 0 for CRC-free) response variables being ${{\boldsymbol{Y}}}_{(1)},\ldots ,{{\boldsymbol{Y}}}_{(K)}$ where

1 $${{\boldsymbol{Y}}}_{(k)}={({Y}_{1k},{Y}_{2k},\ldots ,{Y}_{{n}_{k},k})}^{T},k=1,\ldots ,K.$$

Each of the ${Y}_{{ik}}$, ($i=1,\ldots ,{n}_{k},k=1,\ldots ,K)$ may be related to $G$ groups of genes

2 $${\varPhi }_{{ijk}}=({X}_{i,{j}_{1},k},{X}_{i,{j}_{2},k},\ldots ,{X}_{i,\,{j}_{{g}_{j}},k}),j=1,\ldots ,G,{g}_{j}\ge 0$$

where $i$ is the *i*th individual in the sample, ${g}_{j}$ is the number of genes in $j$ th group. The competing (risk) factor classifier for the *k* th outcome variable is defined as

3 $$\log \left(\frac{{p}_{{ik}}}{1-{p}_{{ik}}}\right)=\max ({\beta }_{01k}+{\varPhi }_{i1k}{\beta }_{1k},{\beta }_{02k}+{\varPhi }_{i2k}{\beta }_{2k},\ldots ,{\beta }_{0{Gk}}+{\varPhi }_{{iGk}}{\beta }_{{Gk}})$$

where ${\beta }_{0{jk}}$’s‘are intercepts, ${\varPhi }_{{ijk}}$ is a $1\times {g}_{j}$ observed vector, ${\beta }_{{jk}}$ is a ${g}_{j}\times 1$ coefficient vector which characterizes the contribution of each predictor to the outcome variable ${{\boldsymbol{Y}}}_{(k)}$ in the *j* th group to the risk, and ${\beta }_{0{jk}}$ + ${\varPhi }_{{ijk}}{\beta }_{{jk}}$ is called the *j* th competing risk factor, i.e., *j* th signature.

***Remark 1***. With ${\beta }_{0{jk}}=-\infty ,j=2,\ldots ,G$, (3) is reduced to the classical logistic regression classifier. It is clear that every component of ${\beta }_{0{jk}}+{\varPhi }_{{ijk}}{\beta }_{{jk}},\,j=1,\ldots ,G$ is a risk factor for a patient to have CRC, and the highest risk is from the largest one, i.e., these risk factors compete against each other to win out to make the final effect, i.e., to determine whether or not a patient has CRC. As such, they are called competing risk factors.

The unknown parameters are estimated from

4 $$(\hat{{\beta }_{(k)}},\hat{S})={\arg }{\min }_{{\beta }_{(k)},{S}_{j}\subset S,j=1,2,\ldots ,G}\mathop{\sum }\limits_{i=1}^{n}[I({p}_{{ik}}\le 0.5)I({Y}_{{ik}}=1)+I({p}_{{ik}} \,>\, 0.5)I({Y}_{{ik}}=0)]$$

where 0.5 is a probability threshold value that is commonly used in machine learning classifiers, $I(.)$ is an indicator function, ${p}_{i}$ is defined in Eq. (3), $S=\left\{\mathrm{1,2},\ldots ,54675\right\}$ is the index set of all genes, ${S}_{1}=\{{1}_{1},{1}_{2},\ldots ,{1}_{{g}_{1}}\}$, ${S}_{2}=\{{2}_{1},\ldots ,{2}_{{g}_{2}}\}$, $\ldots$, ${S}_{G}=\{{G}_{1},\ldots ,{G}_{{g}_{G}}\}$ are index sets corresponding to (2), and $\hat{S}=\{{1}_{1},{1}_{2},\ldots ,{1}_{{g}_{1}};{2}_{1},\ldots ,{2}_{{g}_{2}};\ldots ;{G}_{1},\ldots ,{G}_{{g}_{G}}\}$ is the final gene set selected in the final classifiers.

To introduce sparsity for both the number of variables (genes) and the number of groups (competing factors, signatures) into the model, the following optimization problem with penalties is considered:

5 $$\begin{array}{l}(\hat{\beta },\hat{S},\hat{G})={\text{argmin}}_{\beta ,{S}_{j}\subset S,j=1,2,\ldots ,G}\left\{\right.{(1+{\lambda }_{1}+{\rm{|}}{S}_{u}{\rm{|}})}^{\mathop{\sum }\limits_{k=1}^{K}\mathop{\sum }\limits_{i=1}^{n}[I({p}_{{ik}}\le 0.5)I({Y}_{{ik}}=1)+I({p}_{{ik}} > 0.5)I({Y}_{{ik}}=0)]}\\\qquad\qquad\;\;\,+\,{\lambda }_{2}({\rm{|}}{S}_{u}{\rm{|}}-\frac{{\rm{|}}{S}_{u}{\rm{|}}+G-1}{({\rm{|}}{S}_{u}{\rm{|}}+1)\times G-1})\left.\right\}\end{array}$$

where ${S}_{u}$ is the union set of $\{{S}_{j}{\}}_{j=1}^{G}$, $|\cdot |$ is the cardinality. Tuning parameters ${\lambda }_{1}$ and ${\lambda }_{2}$ are both non-negative. $\frac{|{S}_{u}|+G-1}{(|{S}_{u}|+1)\times G-1}$ is monotone decreasing in both $|{S}_{u}|$ and $G$. Additional properties of this bivariate function are described elsewhere^21^.

***Remark 2***. The S4 property of (5) and its capability to simultaneously classify multiple heterogeneous populations with common variables (genes) make the new competing risk factor classifier different from existing ones.

***Remark 3***. The details of computational steps were described elsewhere^23^ and demo ${\mathrm{Matlab}}^{\circ{\mathrm{R}}}$ codes are publicly available online. Note that Eq. (5) is an optimization problem with extremely high computational complexity, as it’s an integration of integer programming, combinatorial optimization, and continuous optimization. Therefore in practice and for this study, we adopted Monte Carlo approach to solve Eq. (5). Here, we restate the computational guidance for this optimization problem elaborated in our earlier work^23^ with slight modifications according to our setting and references therein.

1. Set G = 1, S_1_ = 3 (or 4, 5). Pre-define sensitivity level sen = 0.6 (or 0.7, 0.8, 0.9) and specificity level spe = 0.90 (or 0.95). The initial selection of pre-defined sen and spe levels may rely on previous literature or researchers’ target.
2. Perform 50,000 (or larger numbers) random draws of sets of S_1_ genes.
3. Evaluate each set of S_1_ genes in Step 2 and calculate the sensitivity and specificity. These genes are recorded if both of their sensitivity and specificity are larger than the pre-specified sen and spe levels. This step helps to filter key genes and reduce the number of genes in scope.
4. If the number of recorded genes in Step 3 is greater than 30 (or 25, 20, depending on the target of gene number shrinking), raise sen and spe values and repeat Step 2 with random draws among recorded genes in Step 3. Repeat Step 3 to further filter the recorded genes.
5. Repeat Step 4 until the number of recorded genes is less than 30 (or 25, 20). These recorded genes are considered as candidate genes.
6. Set G = 3 (or 2,4, 1), S_i_ = 3 (2, 4, 1). Perform 50,000 (or larger numbers) random draws of sets of S_i_ genes among the candidate genes selected in Step 5.
7. Report the best results with the S4 properties.

In this study, we took advantage of a published paper^18^ which studied 1991 genes, and we further reduced the number from 1991 to 9. We conducted a full search to obtain the current results. The procedure is called merging variable selections and common ground seeking (MVS-CGS) and is as follows.

- Choose a dataset^18^.
- For each gene,
  1. Compute its mean within CRCs;
  2. Compute its mean within non-tumors;
  3. Compute its standard deviation within CRCs;
  4. Compute its standard deviation within non-tumors;
  5. Compute its Sharpe ratio, i.e., mean/standard deviation, within CRCs;
  6. Compute its Sharpe ratio within non-tumors;
  7. Compute the absolute relative mean change of means of CRCs over non-tumors;
  8. Compute the absolute relative standard deviation change of standard deviations of CRCs over non-tumors;
  9. Compute the absolute relative Sharpe ratio change of Sharp ratios of CRCs over non-tumors.
- Sort the changes within mean, standard deviation, and Sharpe ratio, respectively.
  1. Keep genes from the bottom 1% of the sorted changes related to means;
  2. Keep genes from the top 15% of the sorted changes related to standard deviations;
  3. Keep genes from the top 5% of the sorted changes related to Sharpe ratios.
- Set a threshold 0.5 for genes kept using Sharpe ratio.
  1. For each gene in the kept set using Sharpe ratio,
    1. Compute the mean (Tm) of Sharpe ratios within CRCs;
    2. Compute the mean (Nm) of Sharpe ratios within non-tumors;
    3. If Tm > Nm and the proportion of CRC patients whose gene expression values are greater than the maximum of gene expression values of non-tumors, keep this gene as a final candidate.
    4. If Tm < Nm and the proportion of CRC patients whose gene expression values are smaller than the minimum of gene expression values of non-tumors, keep this gene as a final candidate.
- Set a threshold 0.7 for genes kept using mean, do the same as above.
- Set a threshold 0.8 for genes kept using standard deviation, do the same as above.
- Now we have a set of genes with which we can run a full search.
- If we want to further reduce the size of the final gene set, we can take squared transformation of the data (or other transformations), do the above procedure to get a new set of genes, than take the common genes as the final gene set.

## Data Availability

The first public dataset was obtained from the NCI’s Genomic Data Commons (GDC) and included 288 CRC samples and 41 normal controls. This RNA-seq study was performed in the TCGA Colon Cancer (COAD) cohort using the Illumina HiSeq 2000 RNA Sequencing platform^46^. Gene expression values were ${\log }_{2}$(norm count+1) transformed. The “normal” samples were adjacent nontumor colorectal tissues. The data link is https://gdc-hub.s3.us-east-1.amazonaws.com/download/TCGA-COAD.htseq_counts.tsv.gz. We noted that this dataset was expanded from 349 samples to 512 samples since we first downloaded from the website. We used 349 samples in our initial data analysis. In our analysis of the second dataset, we used 512 samples which had different measurements from the first dataset (see below). The second public dataset was also obtained from the NCI’s GDC and included 471 CRC samples and 41 normal controls (a total of 512 samples). This RNA-seq study was performed in the TCGA COAD cohort using the Illumina HiSeq 2000 RNA Sequencing platform^46^. The expression values were normalized with ${\log }_{2}$(Fragments Per Kilobase of transcript per Million mapped reads (FPKM) + 1). In our computation, the expression values were further transformed using a natural logarithm operator. The “normal” samples were adjacent normal colon/rectal tissues. The data link is: https://gdc-hub.s3.us-east-1.amazonaws.com/download/TCGA-COAD.htseq_fpkm.tsv.gz. The first dataset and the second dataset had measurement heterogeneity which may cause batch effects when applying classical statistical models and classifications. Our newly proposed max-logistic competing regression can overcome the batch effects by logarithm transformation of the second dataset (see below for details). The third public dataset (GEO Accession: GSE39582) was obtained from a study performed in Europe using the Affymetrix Human Genome U133 Plus 2.0 Array platform^11^. This dataset included 566 CRC samples and 19 normal controls with 54,675 genes/transcripts. The expression values were derived from ${\log }_{2}$(normalized intensity signal). This dataset included frozen tissue of primary colorectal adenocarcinoma and its “normal” samples were frozen tissue of non-tumoral colorectal mucosa. The fourth public dataset (GEO Accession: GSE9348) was obtained from a study performed in a Han Chinese CRC cohort including 82 age-, ethnicity- and tissue-matched healthy controls using the Affymetrix U133 Plus 2 array^47^. The patients were classified as early-stage CRC (Stage 1 or 2). Gene expression values were calculated using the MAS5 algorithm. This dataset included tumor tissue collected and archived within 30 minutes after surgery, and its “normal” was colonic mucosa collected and archived within 30 minutes after biopsy. The fifth public dataset (GEO Accession: GSE18105) was obtained from a study performed in a Japanese cohort containing 77 CRC samples and 17 paired samples from adjacent nontumor tissues^48^. The patients were classified as stages 2 or 3 CRC. Gene expression values were derived from RMA signal intensities. The sixth public dataset (GEO Accession: GSE41258) was obtained from a study performed in an Israel population containing 299 samples, including 180 CRC, 46 polyps, 43 normal colon, 21 liver metastases, and 9 lung metastases^49^. Data were normalized using the PLIER algorithm and batch corrected, then Lowess normalized signals. To validate the results of the discovery analysis, we collected a sample at Sun Yat-sen University Cancer Center in Guangzhou, China, which included 45 CRC samples and 47 normal controls collected from adjacent nontumor colonic tissues (seventh dataset). The genes identified in the discovery analysis were validated using real-time quantitative RT-PCR with the TaqMan Gene Expression assays (Applied Biosystems, Inc.). In addition, the patients’ age, sex, TNM tumor stage, and histologic grade of the tumor were included in the analyses. Regarding this new data collection, the following procedure had been utilized. • Complying with the ‘Guidance of the Ministry of Science and Technology (MOST) for the Review and Approval of Human Genetic Resources’, this project obtained approval from the institutional ethics committee (IEC) of Sun Yat-sen University Cancer Center. Experimental procedures and data collection involving Chinese patients were conducted in China with the participation of Chinese co-authors. • This study obtained approval from the institutional ethics committee (IEC) of Sun Yat-sen University Cancer Center, adhering to the principles of the Declaration of Helsinki. All experimental procedures were conducted in compliance with the guidelines and regulations for the protection of human subjects. Informed consent was obtained from all patients prior to their participation in the study. • No sequencing experiment was performed using the clinical samples collected from Sun Yat-sen University Cancer Center in Guangzhou, China. • Participants provided written informed consent to take part in all studies conducted by Sun Yat-sen University Cancer Center. • The four gene expression values are made available for download from the datalink specified in Code availability. Three additional public datasets (i.e., eighth, ninth and tenth) were included in this study to further validate the findings and to fit our models into preclassified CMS. The eighth public dataset was also obtained from the NCI’s GDC and included 167 rectal cancer samples and 10 normal controls (adjacent normal rectal tissues). The data link is: https://gdc-hub.s3.us-east-1.amazonaws.com/download/TCGA-READ.htseq_fpkm.tsv.gz. Data from the same sample but from different vials/portions/analytes/aliquotes was averaged; data from different samples was combined into genomicMatrix. This RNA-seq study was performed in the TCGA COAD cohort using the Illumina HiSeq 2000 RNA Sequencing platform^46^. The expression values were normalized with ${\log }_{2}$(Fragments Per Kilobase of transcript per Million mapped reads (FPKM) + 1). The ninth public dataset (GEO Accession: GSE103512) contained formalin-fixed and paraffin-embedded normal and tumor tissues of four cancer types, in which colon cancer was included. The platform used was Affymetrix HT-U133plus-2-PM microarrays^50^. In this study, 57 CRC samples and 12 matched normal colon samples were analyzed^50^. The tenth public dataset (GEO Accession: GSE156451) contained tumor tissues from 72 CRC and adjacent nontumor colorectal tissues from a Chinese population. The platform was Illumina NovaSeq 6000 (Homo sapiens)^17^.

## Code availability

A MATLAB^®^ R13 demo code for solving Eq. (4) (λ2 = 0) and readme files are also available. The final dataset organized from the original datasets and our computer program generated datasets and formulas are also available at https://pages.stat.wisc.edu/~zjz/POsubmissions.zip for review. Readers can also make requests by sending emails to the corresponding authors if the link is not working.
